# Above-Ground Dimensions and Acclimation Explain Variation in Drought Mortality of Scots Pine Seedlings from Various Provenances

**DOI:** 10.3389/fpls.2016.01014

**Published:** 2016-07-07

**Authors:** Hannes Seidel, Annette Menzel

**Affiliations:** ^1^Professorship of Ecoclimatology, Department of Ecology and Ecosystem Management, TUM School of Life Sciences Weihenstephan, Technische Universität MünchenFreising, Germany; ^2^Institute for Advanced Study, Technische Universität MünchenGarching, Germany

**Keywords:** die-back, climate change, experiment, drought, warming, acclimation, above-ground biomass, height

## Abstract

Seedling establishment is a critical part of the life cycle, thus seedling survival might be even more important for forest persistence under recent and future climate change. Scots pine forests have been disproportionally more affected by climate change triggered forest-dieback. Nevertheless, some Scots pine provenances might prove resilient to future drought events because of the species’ large distributional range, genetic diversity, and adaptation potential. However, there is a lack of knowledge on provenance-specific survival under severe drought events and on how acclimation alters survival rates in Scots pine seedlings. We therefore conducted two drought-induced mortality experiments with potted Scots pine seedlings in a greenhouse. In the first experiment, 760 three-year-old seedlings from 12 different provenances of the south-western distribution range were subjected to the same treatment followed by the mortality experiment in 2014. In the second experiment, we addressed the question of whether acclimation to re-occurring drought stress events and to elevated temperature might decrease mortality rates. Thus, 139 four-year-old seedlings from France, Germany, and Poland were subjected to different temperature regimes (2012–2014) and drought treatments (2013–2014) before the mortality experiment in 2015. Provenances clearly differed in their hazard of drought-induced mortality, which was only partly related to the climate of their origin. Drought acclimation decreased the hazard of drought-induced mortality. Above-ground dry weight and height were the main determinants for the hazard of mortality, i.e., heavier and taller seedlings were more prone to mortality. Consequently, Scots pine seedlings exhibit a considerable provenance-specific acclimation potential against drought mortality and the selection of suitable provenances might thus facilitate seedling establishment and the persistence of Scots pine forest.

## Introduction

Numerous forest-diebacks have been observed in the last few decades triggered by recent climate change-related drought stress and heat spells (Allen et al., 2010) covering a wide range of climate zones from boreal to tropical regions. Risk of forest mortality may increase in the future as climate models project an increase of temperature and a decrease in precipitation. Thus, drought severity might be exacerbated by rising temperatures (Kirtman et al., 2013). Drought-induced tree mortality may have adverse effects on forest structure and ecological communities, ecosystem function and services as well as biosphere–atmosphere interactions (reviewed, e.g., in Anderegg et al., 2012).

Acclimation (adjustment to environmental changes) is a key process to resist re-occurring stress events (Kozlowski and Pallardy, 2002). Mechanisms involved in drought and warming acclimation comprise molecular, physiological, and structural adjustments. Molecular adjustment against drought typically enhances gene expression pathways for production of molecules, such as abscisic acid (ABA), proline, and soluble sugars, linked to the maintenance of turgor and cell integrity (Peñuelas et al., 2013). ABA additionally inhibits cell expansion and thus reduces growth (Lambers et al., 2008). Warming acclimation on the molecular level increases the synthesis of heat shock and anti-stress proteins (Peñuelas et al., 2013). Physiological drought acclimation comprises changes in resource allocation (Callaway et al., 1994; Peña-Rojas et al., 2005; Poorter et al., 2012), a decrease of photosynthetic activity (Rennenberg et al., 2006) and an increase of water use efficiency (Brodribb and Hill, 1998; Lloret et al., 2004a). Structural acclimation processes toward water stress are the reduction of total leaf area by leaf size reduction (Kubiske and Abrams, 1992; Martínez-Vilalta et al., 2009; Günthardt-Goerg et al., 2013) or leaf shedding (Ogasa et al., 2013), the adjustment of the hydraulic system by altering xylem conduit dimensions (Eilmann et al., 2009; Bryukhanova and Fonti, 2012), leaf/sapwood area ratio and leaf-specific hydraulic conductivity (Martínez-Vilalta et al., 2009). Furthermore, reduction of above-ground biomass might be linked to increased drought resistance (Alía et al., 2001; Valladares et al., 2007).

The seedling stage is an ontogenetic phase that is very drought sensitive and prone to mortality (Hanson et al., 2001; Lloret et al., 2004b) likely due to lower carbohydrate reserves (Niinemets, 2010), smaller rooting volume and lower rooting depth than mature trees (Cavender-Bares and Bazzaz, 2000). In general, proposed mechanisms of drought-induced tree mortality are carbon starvation caused by depletion of carbohydrate pools (Galiano et al., 2011; Adams et al., 2013; Mitchell et al., 2013), hydraulic failure of xylem conduits impairing water transport (Anderegg and Anderegg, 2013; Mitchell et al., 2013; Salmon et al., 2015), and failure of phloem transport affecting carbon translocation (Sala et al., 2010; McDowell et al., 2011; Adams et al., 2013). Survival rates of seedlings under drought were found to depend on various traits. The survival of several Mediterranean tree species is negatively correlated with total plant dry mass, total leaf area and positively correlated to leaf area ratio and shoot–root ratio across species (Valladares and Sánchez-Gómez, 2006). Thus, trait characteristics indicating a higher transpiring surface are linked to higher mortality rates. Tree size in general increases the hazard of drought-induced mortality (Bennett et al., 2015) which might be explained by an increased risk of hydraulic dysfunction with increasing tree height and greater leaf area (McDowell and Allen, 2015).

Successful and adequate seedling establishment is essential for sustainable forest cover and production. Decreasing seedling survival under climate change may hamper forest regeneration and thus result in alternative forest communities or even non-forest ecosystems (Anderson-Teixeira et al., 2013). Bussotti et al. (2015) proposed three mechanisms of forest adaption to future environmental conditions: persistence by acclimation and phenotypic plasticity, evolution or local adaptation, and migration or substitution of tree species. If natural migration cannot keep pace with the rate of climate change, assisted migration of more adapted alternative tree species, but also suitable provenances within the same species, might support timely adaptation. Several studies have demonstrated provenance differences in sensitivity and response to reduced water availability, indicated, e.g., by differences in shoot length, diameter increment, and stomatal conductance of *Fagus sylvatica* (Rose et al., 2009; Thiel et al., 2014; Knutzen et al., 2015), in height and dry weight of *Picea abies* (Modrzyński and Eriksson, 2002), and in stomatal conductance, transpiration rates, leaf hydraulic conductance, osmotic potential, loss of hydraulic conductivity, and water-use efficiency of *Pinus halepensis* (Tognetti et al., 1997; Klein et al., 2013). However, studies on provenance-specific drought mortality are rare. Mortality rates were evaluated for central and marginal provenances of *F. sylvatica* (Thiel et al., 2012), provenances of *Quercus pubescens* (Wellstein and Cianfaglione, 2014), *Pinus ponderosa* provenances from origins differing in summer drought (Cregg, 1994) and central provenances of *Pinus sylvestris* (Cregg and Zhang, 2001), but only *P. ponderosa* provenances differed in drought survival, although not significantly.

More than one-third of the forest diebacks reported by Allen et al. (2010) are linked to Scots pine (*P. sylvestris* L.) forests although this species is considered to be drought resistant (Ellenberg, 1988). Scots pine has an extensive latitudinal (Spain to Scandinavia) and longitudinal (Spain to the far east of Russia) distribution range covering various climate zones from Mediterranean to boreal habitats (Boratynski, 1991). The huge distribution of this species favors local adaptation of provenances to contrasting environmental conditions (Boratynski, 1991; Reich and Oleksyn, 2008). The southernmost rear age of the distribution range in Spain and Italy is composed of post-glacial relict populations (Boratynski, 1991) which might be of special importance for provenance-based assisted migration due to high levels of genetic differentiation (Hampe and Petit, 2005) or even adaptations to heat and drought (Alía et al., 2001). Scots pine is an isohydric species which aims at minimizing water loss by tight stomatal control (Irvine et al., 1998) as well as by adjustment of leaf/sapwood area ratio, leaf-specific hydraulic conductivity, total leaf area, and conduit size (Sterck et al., 2008; Martínez-Vilalta et al., 2009). This drought avoiding strategy by closing stomata is implemented at the cost of reduced photosynthetic carbon gain (Mitchell et al., 2013), thus the risk of drought-induced mortality might be especially observed under moderate but long lasting droughts. Regarding local adaptations, Scots pine provenances show differing drought responses in seedling establishment (Richter et al., 2012), in shoot diameter and height increment (Taeger et al., 2013a, 2015), and drought resistance (Taeger et al., 2013b).

However, there is a lack of knowledge how drought acclimation and provenance effects (adaptation) may impact mortality rates of *P. sylvestris* which would allow optimizing the provenance choice for assisted migration. In order to address this issue, we conducted two drought mortality experiments with potted Scots pine seedlings. The first mortality experiment in 2014 was based on 12 different Scots pine provenances from its south-western distribution in Europe. The second mortality experiment was conducted in 2015 and investigated seedlings of three provenances that had been acclimated to different seasonal drought events and temperature regimes before. Drought acclimation included a spring and a summer drought in 2013 and a spring drought in 2014. All drought acclimation treatments were applied within two temperature regimes, namely ambient air temperature and passively elevated temperature in a greenhouse. We hypothesized that (1) the hazard of mortality induced by a severe drought treatment differs across provenances, (2) acclimation by previous drought events and elevated temperature decreases the hazard of drought-induced mortality, and (3) the hazard rate depends on the climate at the provenances’ origin.

## Materials and Methods

### Plant Material

Scots pine seedlings originated from 12 provenances distributed along their south-western distribution. Climatic conditions at the origin of the seeds range between ∼3 and 11°C annual mean temperature and ∼600 and 1100 mm of annual precipitation comprising western Mediterranean (Spain, France, and Italy) and continental (Switzerland, Germany, Poland, Hungary, and Bulgaria) sites (**Table 1**). Plants were grown from seeds in a nursery in south-eastern Germany in 2011. All seeds were collected from autochthonous populations, except for Alpenkiefer from Germany and Plantage Pornoapati from Hungary which were obtained from seed orchards. In 2012, seedlings were brought to the Gewächshauslaborzentrum (GHL) near Freising, Germany, where they were potted into 3 l pots containing peat substrate. Seedling treatments/acclimation and provenances studied differed between the first mortality experiment conducted in 2014 and the second one conducted in 2015 as explained in the next chapters.

**Table 1:** Origin of seeds used in the mortality experiments.

Provenance abbreviation	Provenance	Country	Latitude	Longitude	Altitude (m)	*T* (°C)	PPT (mm)
PL9	Suprasl, PL	Poland	53°15′N	23°23′E	181	6.6	584
D8	Mittel-/Ostdt. Tiefland, D	Germany	53°04′N	13°29′E	75	8.5	574
D6	Hauptsmoorwald, D	Germany	49°51′N	10°58′E	250	8.8	646
D7	Alpenkiefer, D	Germany	47°30′N	11°20′E	1150	3.2	1106
HU14	Plantage Pornoapati, HU	Hungary	47°20′N	16°28′E	300	10.0	598
CH5	Wallis, CH	Switzerland	46°18′N	07°39′E	900	7.2	1025
I4	Emilia Romagna, I	Italy	44°30′N	10°27′E	460	10.8	888
F12	Mont Ventoux, F	France	44°10′N	05°16′E	1600	3.9	1166
F3	Prealpes du Sud, F	France	43°45′N	06°40′E	1185	7.6	955
ES1	Alto Ebro, ES	Spain	42°59′N	03°17′W	860	10.1	940
BG10	Garmen, BG	Bulgaria	41°43′N	23°54′E	1300	6.6	649
ES2	Montes Universales, ES	Spain	40°28′N	01°53′W	1670	7.8	644


*Annual mean temperature (T), annual sum of precipitation (PPT) are shown for the period 1950–2000 (WorldClim data base; Hijmans et al., 2005). All seeds except the provenances HU14 and D7 (seed orchards) are from autochthonous stands. Provenances are arranged in decreasing order of latitude.*

### Mortality Experiment 2014

**FIGURE 1:**
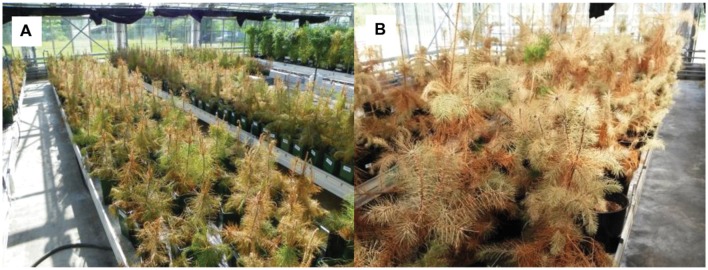
**Scots pine seedlings of different provenances in the mortality experiments.** **(A)** After 83 days of total withholding of water in 2014 (mortality experiment from March 26 to July 1, 2014). **(B)** After 75 days of total withholding of water in 2015 (mortality experiment from May 22 to August 31, 2015).

Seedlings used in the first mortality experiment in 2014 comprised all 12 provenances listed in **Table 1.** In contrast to the second mortality experiment (see Mortality Experiment 2015 and Acclimation Treatments), they had not experienced any prior acclimation treatment and had been exclusively grown in the greenhouse. In total, 760 individuals were examined, but numbers of individuals per provenance varied between 31 and 187 individuals per provenance (**Table 2**). They were randomly arranged on three tables next to each other in a greenhouse (**Figure 1A**). All pots got well watered manually until the lethal drought treatment started by withholding irrigation (March 26 to July 1, 2014).

**Table 2:** Number of replicates, percentage of mortality after the lethal drought period, above-ground dry weight and height for different provenances, drought treatments and buildings (temperature regimes) in the mortality experiments in 2014 and 2015.

Provenance	*N*	Percent mortality	Weight ±*SD* (g)	Height ±*SD* (mm)
**Mortality experiment 2014 (March 26 to July 1)**				
Suprasl, PL	50	96.00	27.61 ± 6.74^ab^	/
Mittel-/Ostdt. Tiefland, D	41	92.68	30.46 ± 7.10^ac^	/
Hauptsmoorwald, D	31	96.77	28.00 ± 5.67^abd^	/
Alpenkiefer, D	54	98.15	34.82 ± 6.54^c^	/
Plantage Pornoapati, HU	52	98.08	31.78 ± 7.33^bc^	/
Wallis, CH	94	97.87	22.39 ± 9.26^e^	/
Emilia Romagna, I	42	80.95	30.59 ± 7.35^ac^	/
Mont Ventoux, F	57	98.25	27.42 ± 7.05^ab^	/
Prealpes du Sud, F	47	85.11	26.57 ± 8.48^aef^	/
Alto Ebro, ES	56	96.43	25.67 ± 6.99^ae^	/
Garmen, BG	49	89.80	30.71 ± 7.64^bcf^	/
Montes Universales, ES	187	94.12	23.33 ± 8.77^de^	/
**Mortality experiment 2015 (May 22 to August 31)**				
Suprasl, PL	52	98.08	153.88 ± 55.05^a^	1030.85 ± 260.65^a^
Alpenkiefer, D	43	100.00	182.23 ± 47.98^b^	956.19 ± 141.67^a^
Mont Ventoux, F	44	100.00	133.93 ± 38.61^a^	734.84 ± 158.60^b^
**Drought treatment 2013 (spring–summer)**				
Control–control	36	100.00	168.39 ± 50.52^a^	1001.28 ± 246.49^a^
Control–drought	34	100.00	153.89 ± 47.26^a^	923.21 ± 215.58^a^
Drought–control	37	100.00	156.64 ± 51.18^a^	862.81 ± 222.80^a^
Drought–drought	32	96.88	145.11 ± 56.61^a^	865.44 ± 233.59^a^
**Drought treatment 2014 (spring)**				
Control	58	100	190.65 ± 46.51^a^	991.07 ± 244.67^a^
Drought	81	98.77	131.49 ± 39.39^b^	858.90 ± 211.71^b^
**Building 2012–2014**				
Vegetation Hall	65	100	173.10 ± 44.23^a^	1014.23 ± 191.79^a^
Greenhouse	74	98.65	141.87 ± 53.23^b^	826.05 ± 234.34^b^


*Height was not measured in 2014. In the mortality experiment 2015, the seedlings were acclimated in different drought and temperature regimes during 2013 and 2014. The drought treatment in 2013 was applied in two phases with a spring and/or a summer drought from March 22 to June 14 and July 10 to August 21, respectively. The drought treatment in spring 2014 lasted from March 23 to June 23. Seedlings were acclimated in two temperature regimes, in a vegetation hall (ambient temperature) and a greenhouse (elevated temperature by passive warming). Provenances are arranged in decreasing order of latitude. Mean values for above-ground dry weight and height are given with respective standard deviation (SD). Group differences were calculated comparing contrasts with the Tukey’s range test. Values sharing the same letter are not significantly different.*

### Mortality Experiment 2015 and Acclimation Treatments

In total, 139 seedlings were studied in the second mortality experiment in 2015. They comprised three provenances (Alpenkiefer, D; Mont Ventoux, F; Suprasl, PL) with 43, 44, and 52 individuals, respectively (**Table 2**). These seedlings were remnants of an extensive seasonal drought and warming experiment on more than 1000 individuals from 10 European Scots pine provenances. Out of these, three provenances were selected in order to provide sufficient sample sizes and to cover contrasting climatic conditions/seasonal precipitation patterns (**Table 1**, **Supplementary Figure S1**). Prior to this second mortality experiment seedlings were subjected to two different temperature acclimation treatments, and within each they were exposed to drought acclimation treatments in 2013 and 2014 in order to study the influence of carry-over effects on mortality (see description of acclimation treatments below). In March 2014, seedlings were replanted from 3 l pots into 20 l pots containing peat substrate of identical composition as before. All 139 plants were put together in the greenhouse in December 2014 (**Figure 1B**), were randomly distributed on three tables and got well watered till the start of the mortality experiment in May 2015. During the lethal mortality experiment, irrigation was totally intermitted from May 22 to August 31, 2015.

#### Temperature Acclimation

Seedlings were grown under two temperature regimes from mid of 2012 till end of 2014 (**Supplementary Figure S2**). During this period 65 of the 139 seedlings were placed in a vegetation hall (glass-roofed building with open sidewalls), and 74 seedlings were placed in the greenhouse. Temperatures in the vegetation hall were similar to ambient conditions, but passive warming of the greenhouse increased temperatures. Within each building, air temperature and relative humidity (RH) were recorded in 10-min intervals with a temperature/RH data logger (HOBO U23 Pro v2, Hobo^®^, Onset Computer Corporation, Bourne, MA, USA). The vapor pressure deficit (VPD) was calculated using air temperature and RH as input variables after Allen et al. (1998) as a measure of atmospheric dryness. The mean temperature difference during 2013 and 2014 between the vegetation hall and the greenhouse was 3.0°C (**Supplementary Figure S2B**); however, the difference was more pronounced in frost periods (defined as periods with days having temperatures below 0°C), i.e., 5.7°C (January 1 to April 8, 2013), 4.0°C (November 12, 2013 to April 17, 2014) and 5.8°C (December 8–11, 2014) than in frost-free summer periods (1.3°C in 2013 and 2.3°C in 2014). The daily means of VPD were in general higher in the greenhouse than in the vegetation hall with some exceptions during the whole acclimation period (**Supplementary Figure S2D**). The overall mean VPD during 2013 and 2014 was 0.16 kPa higher in the greenhouse than in the vegetation hall.

#### Drought Acclimation in 2013 and 2014

The drought acclimation comprised three drought periods in total (two in 2013, one in 2014), identical within each temperature regime, and accompanied by respective well-watered control groups (**Supplementary Figure S3**). In 2013, the drought was applied in two separated periods with a spring and/or a summer drought from March 22 to June 14 and July 10 to August 21, respectively. In both periods, automated irrigation was stopped and thereafter, soil moisture was adjusted to oscillate around the permanent wilting point by adding small amounts of water when necessary. The permanent wilting point (pF 4.2) corresponded to 12 Vol% soil moisture derived from water retention curves following the pressure plate method by Richards (1941). Thus, drought treated seedlings had to survive near the limit (permanent wilting point) for around 5 weeks in spring and for around 4 weeks in summer 2013 and got well-watered in between. The control and the three drought treatment groups (spring drought, summer drought, spring and summer drought 2013) comprised 32–37 individuals (**Table 2**).

These individuals of 2013 were split to a spring drought and well-watered control group in 2014, all within their respective temperature regime. This drought acclimation treatment lasted from March 23 to June 23 by totally withholding irrigation. Soil moisture fell below the permanent wilting point for around 5 weeks. Fifty-eight individuals belonged to the watered control group and 81 individuals were part of the drought acclimation group.

Soil moisture was monitored twice a week in the afore mentioned overarching experiment using a hand-held soil moisture sensor (UMP1, Umwelt-Geräte-Technik GmbH, Müncheberg, Germany) on 240 pots equally spread across provenances and treatments (**Supplementary Figure S3**).

### Mortality, Above-Ground Dry Weight and Height Assessment and Meteorological Conditions during the Mortality Experiments

Mortality assessment was done with a knife carefully scratching the bark. The seedlings were classified as alive if the cambium tissue underneath the bark was green and classified as dead when this tissue was brownish. Evaluation of mortality was done on six dates in 2014 from April 28 to July 1 (day 33, 54, 62, 76, 82, and 97 after the lethal drought started) and almost once a week between June 2 and August 31 in 2015 (on day 12, 19, 26, 32, 40, 47, 54, 61, 68, 75, 82, 88, and 102 after initiation of the lethal drought treatment). In 2015, the height of seedlings was measured at the beginning of the experiment from the substrate surface to the terminal tip using a folding rule. After each mortality experiment, above-ground biomass was harvested and oven dried at 60°C for 48 h to assess total above-ground dry mass.

Meteorological conditions were variable between the mortality experiments conducted in 2014 (March 26 to July 1) and in 2015 (May 22 to August 31). During the study periods mean temperature, mean RH, and mean VPD were 16.6 and 24.5°C; 62.5 and 54.7%; and 0.9 and 1.6 kPa in 2014 and 2015, respectively. Variables ranged from 2.9 to 39.1°C, 21.9 to 93%, and 0.1 to 5.3 kPa in 2014; and from 11.2 to 41.6°C, 18.8 to 88.5%, and 0.2 to 6.3 kPa in 2015, clearly indicating that the lethal drought stress in summer 2015 was higher than in spring 2014.

### Statistics

#### Above-Ground Dimensions (Seedling Height and Above-Ground Dry Weight)

The effect of provenances and acclimation treatments on seedlings’ above-ground dry weight and height was evaluated separately using linear models (stats, R Core Team, 2015) in R 3.2.2. In the case of analyzing above-ground biomass in the mortality experiment in 2014, just provenance (12 different provenances) served as an explanatory variable. For analyzing above-ground biomass and height in the mortality experiment in 2015, explanatory variables were provenance (three different ones), drought acclimation treatment 2013 (only spring drought, only summer drought, spring and summer drought, and control), drought acclimation treatment 2014 (spring drought, control) and building (vegetation hall, greenhouse). Provenance, drought acclimation treatment and temperature acclimation treatment (building) were incorporated in the models as factorial dummy variables; more precisely, as the presence or absence of drought and the affiliation to vegetation hall (ambient temperatures) or greenhouse (elevated temperatures).

#### Survival Analysis

Survival analysis was conducted using a Cox proportional hazards regression model (survival package in R; Therneau and Grambsch, 2000). This type of model calculates the hazard ratio (HR) that is the probability of a death event in the treatment group in relation to the probability in the reference group. The covariates provenance and above-ground dry weight as well as their two-way interaction were added to analyze mortality of Scots pine seedlings in the mortality experiment 2014.

Two different models were constructed to analyze mortality and the effect of covariates on the drought hazard in the 2015 experiment. The first model contained the covariates provenance, drought treatments in 2013, drought treatment in 2014, temperature regime and the two-way interactions of provenance with drought treatments in 2013, drought treatment in 2014 and temperature regime (so called factorial model). The second model to analyze the 2015 mortality experiment included, in addition to the covariates and two-way interactions of the first model, seedling height and above-ground dry weight (so called continuous model). Provenance, drought acclimation treatment and temperature acclimation treatment were incorporated in the models as factorial dummy variables as explained above. Since above-ground biomass and height varied significantly between levels of provenances and hardening treatments (**Table 2**, see **Supplementary Tables S1** and **S2** for the summary output of linear models), weight and height were centered on respective group means to avoid confounding effects of covariates with tree dimension effects.

All survival models were simplified by stepwise excluding covariates/interactions that did not improve the models explanatory power with the Anova function (car, Fox and Weisberg, 2011). The proportional hazard assumption was checked by examining diagnostic plots and with the cox.zph function (survival; Therneau and Grambsch, 2000).

Pairwise comparisons in the linear and Cox proportional hazard models were done using the glht function (multcomp, Hothorn et al., 2008) comparing contrasts with the Tukey’s range test and the false discovery rate method was applied to correct *p*-values for multiple comparisons.

## Results

### Seedling Dimensions

Seedling dimensions (above-ground dry weight and height) varied across provenances and acclimation treatments (**Table 2**; see also **Supplementary Tables S1** and **S2** for detailed summary output of models). In the mortality experiment in 2014, the provenance with the largest mean above-ground dry biomass was the Alpenkiefer from Germany with 34.82 g, being significantly heavier than the provenances Wallis, Hauptsmoorwald, Alto Ebro, Montes Universales, Mont Ventoux, Prealpes du Sud, and Suprasl. The provenance from Wallis had the lowest above-ground dry weight (22.39 g) and was significantly different from all other provenances except Alto Ebro, Montes Universales, and Prealpes du Sud. Significant differences across provenances with intermediate weight could be also found (**Table 2**). Both provenances from Spain (Alto Ebro, Montes Universales) had a lower above-ground biomass than Garmen, Plantage Pornoapati, Mittel-/Ostdt. Tiefland, and Emilia Romagna. Additionally, Montes Universales was lighter than Mont Ventoux and Suprasl, and Plantage Pornoapati was heavier than Prealpes du Sud.

Provenances used in the experiment in 2015 differed significantly in mean above-ground dry weight and mean height. The seedlings of Alpenkiefer (182.23 g) were heavier than the pines of Suprasl (153.88 g) and Mont Ventoux (133.93 g). The smallest mean height was scored for the provenance Mont Ventoux (734.84 mm) being significantly smaller than Alpenkiefer (956.19 mm) and Suprasl (1030.85 mm). Regarding the acclimation treatments (**Table 2**) no effect of the seasonal drought events in 2013 could be detected neither on mean above-ground biomass at the end of the experiment in August 2015 nor on seedling height. The drought in 2014 significantly affected mean above-ground dry biomass (190.65 g vs. 131.49 g) and mean seedling height (991.07 vs. 858.9 mm). Pines grown in the vegetation hall from 2012 to 2014 were significantly heavier (173.10 vs. 141.87 g) and taller (1014.23 vs. 826.05 mm) than those grown in the greenhouse.

### Mortality 2014

#### Mortality Rates and Influence of Above-Ground Dry Weight

**FIGURE 2:**
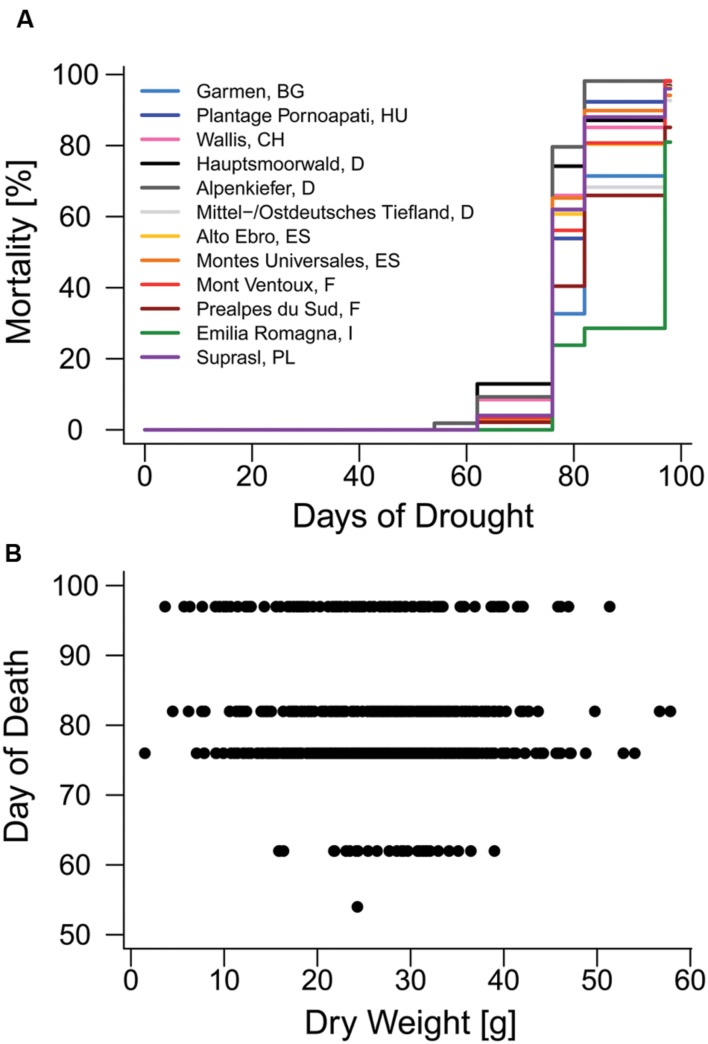
**Mortality in 2014.** **(A)** Mortality rates of provenances during the mortality experiment. **(B)** Relationship between above-ground dry weight and the day when mortality was observed on one of the six observation days (day of death).

The first mortality events of Scots pine seedling were observed on the 54th day (second observation day) of the 97-day drought treatment in 2014 and most diebacks (415 out of 760) were detected on the 62nd day (third observation day) after the drought started (**Figure 2A**, **Supplementary Figure S4A**). Total mortality rates of the 12 provenances used in the experiment (**Table 2**) ranged between 81% (Emilia Romagna, I) and 98% (Mont Ventoux, F; Alpenkiefer, D; Plantage Pornoapati, HU; and Wallis, CH). Variation in mortality rates of seedlings in 2014 could be explained by above-ground dry weight and provenances (**Table 3**, see **Supplementary Table S3** for a detailed model summary). Above-ground dry weight significantly increased the HR by 1.7%/g (*p* < 0.001, **Supplementary Table S4**, **Figure 2B**), thus the hazard of the individual with the highest weight (57.86 g) had an almost 96% higher hazard of mortality than the individual with the lowest weight (1.49 g).

**Table 3:** Analysis-of-variance table evaluating the set of covariates included in final Cox proportional hazards regression models using partial-likelihood ratio test.

Covariates	LR	*df*	*p*
**Mortality experiment 2014**			
Provenance	74.856	11	**<0.001**
Weight	14.693	1	**<0.001**
**Mortality experiment 2015: factorial model**			
Drought treatment 2014	18.94	1	**<0.001**
Provenance	3.22	2	0.200
Building	14.875	1	**<0.001**
Provenance: building	6.667	2	**0.036**
**Mortality experiment 2015: continuous model**			
Height	7.816	1	**0.005**
Weight	7.487	1	**0.006**
Drought treatment 2014	20.238	1	**<0.001**
Provenance	3.535	2	0.171
Building	12.814	1	**<0.001**
Provenance: building	10.488	2	**0.005**


*Bold values indicate significance at a level of 0.05.*

#### Provenance Differences in Mortality Hazard Rates

**FIGURE 3:**
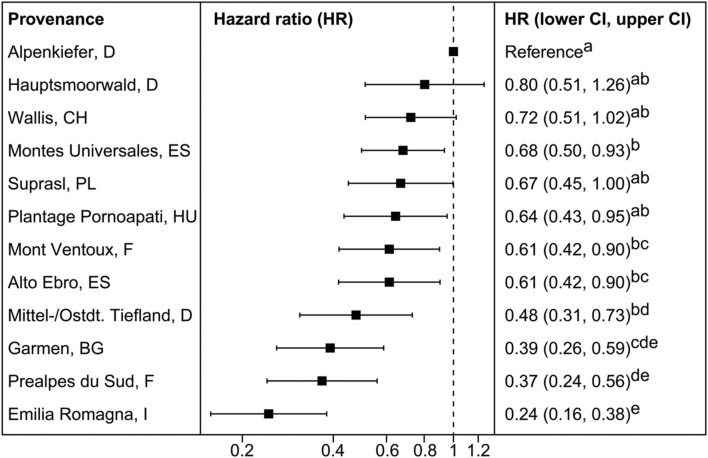
**Mean hazard ratios (HR) and confidence intervals (CI) of provenances investigated in the mortality experiment 2014 in relation to the reference provenance Alpenkiefer (D).** Provenances are arranged in decreasing order of mean HR. Pairwise differences between provenances were calculated comparing contrasts with the Tukey’s range test. HR sharing the same letter are not different at a significance level of 0.05.

Provenances differed significantly in their hazard rate for mortality due to the severe drought event in the experimental set-up (**Figures 2A** and **3**, **Supplementary Table S4**). The provenance from Emilia Romagna (Italy) showed significantly lower hazard rates than all other provenances (*p* < 0.05) with the exception of the provenances Prealpes de Sud (France) and Garmen (Bulgaria), which were the second and third most drought resistant provenances in terms of mortality, respectively. Almost each provenance showed higher hazards than those three, except Mont Ventoux (France) and Alto Ebro (Spain) that did not indicate a higher mortality risk than the provenance Garmen (Bulgaria). Additionally, the provenance Mittel-/Ostdt. Tiefland (Germany) was not at higher risk than the provenances Prealpes de Sud (France) and Garmen (Bulgaria). The most vulnerable provenance in respect to the mortality hazard caused by severe drought was Alpenkiefer (Germany) that was at greater risk than Prealpes de Sud, Mont Ventoux (both France), Alto Ebro, Montes Universales (both Spain), Emilia Romagna (Italy), Garmen (Bulgaria), and Mittel-/Ostdt. Tiefland (Germany).

### Mortality 2015

#### Mortality Rates and Influence of Above-Ground Dimensions

**FIGURE 4:**
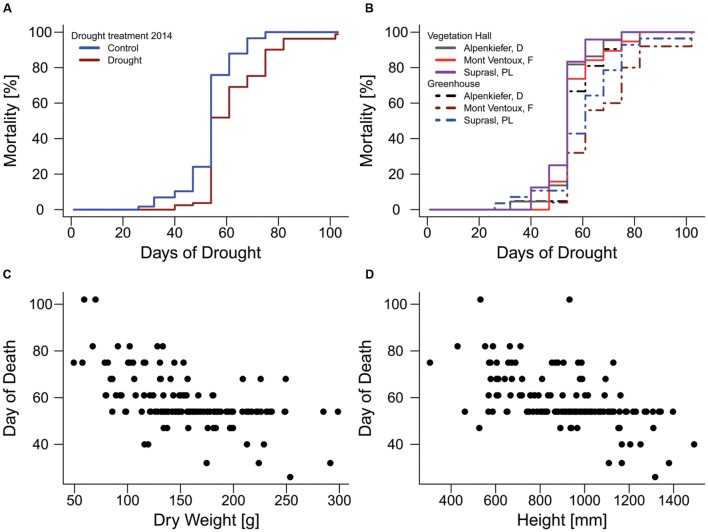
**Mortality rates of pines during the mortality experiment in 2015.** **(A)** Influence of acclimation by the drought treatment in 2014. **(B)** Effects of provenance and temperature acclimation on mortality rates. Relationship of **(C)** above-ground dry weight and **(D)** seedling height with the day when mortality was observed on the 13 observation days (day of death).

The drought mortality experiment in 2015 lasted 102 days. The first dead individual was observed on day 26 after drought initiation and most mortality events were observed on the 54th day of the drought treatment when 69 out of 139 individuals were recorded as dead (**Supplementary Figure S4B**). Total mortality rates (**Table 2**) were almost 100% in all three provenances (Alpenkiefer, D; Mont Ventoux, F; and Suprasl, PL) and for all acclimation groups (drought treatment 2013, drought treatment 2014 and building). Differences in the drought mortality hazard were explained by acclimation induced by the drought treatment in 2014 (*p* < 0.001, **Table 3**) as well as the interaction between provenance and building induced by growing seedlings in the two different buildings, thus temperature regimes, in the so called factorial model (*p* < 0.05, **Table 3**, see **Supplementary Table S5** for a detailed model summary). Adding the covariates above-ground dry weight and height in the so-called continuous model increased model concordance from 0.74 to 0.81 (**Supplementary Tables S5** and **S6**, respectively). Both covariates exhibited a significant influence on the HR (*p* < 0.01, **Table 3**, see **Supplementary Table S6** for a detailed model summary). The HR increased by 0.8%/g above-ground dry weight and by 0.2%/ml height (**Table 4**, **Figures 4C,D**), thus resulting in a doubling of mortality hazard (increase by 199.5%) between the lightest (49.28 g) and the heaviest individual (298.64 g) and in a more than doubling of mortality hazard (increase by 237.4%) between the smallest (305 mm) and the tallest individual (1492 mm).

**Table 4:** Estimated main and interaction effects of the survival model including factorial covariates only (factorial model) and factorial with continuous covariates (continuous model) on hazard of mortality in the experiment 2015.

	Factorial model		Continuous model	
		
	HR	*p*	HR	*p*
**Main effects**				
Drought treatment 2014				
Control/drought	1/0.426	**<0.001**	0.404	**<0.001**
Dimensions				
Height (ΔHR/mm)	/	/	1.002	**0.005**
Weight (ΔHR/g)	/	/	1.008	**0.006**
**Interaction effects (pairwise comparisons)**				
Vegetation hall
D7/F12	1/1.084	0.804	1/1.448	0.409
D7/PL9	1/1.351	0.732	1/1.507	0.409
F12/PL9	1/1.246	0.732	1/1.041	0.901
Greenhouse
D7/F12	1/0.390	**0.007**	1/0.324	**0.001**
D7/PL9	1/0.504	**0.033**	1/0.534	0.055
F12/PL9	1/1.293	0.360	1/1.647	0.081
**Vegetation hall (VegH) vs. greenhouse (GH)**				
D7: GH/VegH	1/1.036	0.909	1/0.847	0.606
F12: GH/VegH	1/2.881	**0.001**	1/3.777	**0.001**
PL9: GH/VegH	1/2.776	**0.001**	1/2.389	**0.004**


*The hazard ratio (HR) shows the probability of a death event in the treatment group in relation to the probability in the reference group or the change of hazard in relation to the change in continuous covariates per one unit (height, weight). Provenance abbreviations are D7 (Alpenkiefer, D), PL9 (Suprasl, PL), and F12 (Mont Ventoux, F). Group differences were calculated comparing contrasts with the Tukey’s range test.*

*Bold values indicate significance at a level of 0.05.*

#### Variation in Mortality Hazard Rates with Acclimation and Provenance

Although drought mortality was almost 100% at the end of the lethal drought experiment in 2015 there were still differences in the mortality hazard among acclimation groups and provenances since the time span till maximum mortality was observed varied between 75 and 102 days (**Figure 4**). The drought treatment in 2014 reduced the mortality hazard by 57.4% in the factorial model and by 59.6% in continuous model compared to the control (**Table 4**, **Figure 4A**). Variation in mortality hazard between provenances depended on the building (temperature regime) in which they were grown till December 2014. Whereas HR did not differ between provenances in the vegetation hall, they did in the greenhouse (**Table 4**, **Figure 4B**). The mortality hazard for Alpenkiefer (Germany) is 61 and 49.6% significantly higher (*p* < 0.05) than for Mont Ventoux (France) and Suprasl (Poland), as estimated with the factorial model. Mont Ventoux (France) and Suprasl (Poland) were not different in their mortality hazard. Adding the covariates above-ground dry mass and seedling height increased the difference in hazard between Alpenkiefer and Mont Ventoux to 67.6% and diminished the hazard between Alpenkiefer and Suprasl to 46.6%, that was not significant anymore at the 5% level (*p* = 0.081). Growing in the vegetation hall till December 2014 increased the hazard of mortality in Mont Ventoux and Suprasl (*p* < 0.01), but not in Alpenkiefer compared to the hazard experienced in the greenhouse (**Table 4**, **Figure 4B**). Hazard in the vegetation hall was 188.1 and 277.7% higher for Mont Ventoux compared to the greenhouse as indicated by the factorial and continuous model, respectively. Suprasl showed a 177.6 and 138.9% higher mortality hazard in the vegetation hall compared to the greenhouse.

## Discussion

Drought mortality varied fundamentally across provenances which was to our knowledge not reported in literature before. Above-ground dimensions had a significant impact on the hazard of drought-induced mortality. Taller and heavier individuals died earlier than individuals which were smaller and had lower weights. Thus, there seems to be a trade-off between growth and drought survival (Alía et al., 2001; Bennett et al., 2015). Acclimating seedlings by drought in the year before the actual drought mortality experiment considerably lowered the mortality hazard. In the further paragraphs, we discuss the link between provenance and above-ground dimensions as well as the provenance and acclimation effects on the mortality hazards.

### Variation of Seedling Dimensions among Provenances and Acclimation Treatments

Seedling dimensions (above-ground dry weight and seedling height) showed considerable variation among provenances and acclimation treatments. Differences in dry weight were consistent for provenances that were monitored in both mortality experiments in 2014 and 2015. Variation in biomass and height across provenances of *P. sylvestris* is well documented in literature. Oleksyn et al. (1992, 1998, 1999, 2000) showed that above-ground biomass and height of seedlings and adult trees had a hump-shaped relationship with the latitude of their origin, central provenances (45–55°N) having higher above-ground biomass and height than the southern (<45°N) and northern provenances (>55°N). This pattern is similar for the latitudinal range (40–53°N) in our study at least for above-ground dry weight, however, it has to be taken into account that the provenance Alpenkiefer is from a seed orchard. Oleksyn et al. (1999) attributed their findings to reduced day length and climate transfer distance in northern provenances and to genetic adaptation to warm and arid environments in southern provenances.

Two of the acclimation treatments (drought treatment 2014, warming treatment building) had a significant impact on seedling dimensions in the mortality experiment 2015. Spring drought in 2014 reduced the seedlings’ above-ground dimensions. Water stress is known to exert a negative influence on the rate of cell wall division and cell expansion (Hsiao and Acevedo, 1974). This is a direct effect of reduced turgor pressure (Hsiao and Acevedo, 1974) or an indirect effect of the suppression by growth regulators such as ABA (Peñuelas et al., 2013). Furthermore, Scots pine increases root–shoot ratio under dry conditions (Richter et al., 2012; Taeger et al., 2015), which might be caused by lower investment in above-ground structures (Alía et al., 2001; Taeger et al., 2013b, 2015).

Seedling above-ground dimensions were smaller when grown in the greenhouse under elevated temperatures from 2012 to 2015. Reich and Oleksyn (2008) reported that height growth of southern Scots pine provenances was negatively correlated with the absolute temperature difference between planting site and origin. Since all the provenances in our study belong to the geographical group which Reich and Oleksyn (2008) classified as southern provenances (≤53°N) this may explain the finding of decreased seedling height at elevated temperature in the greenhouse and might also contribute to the lower above-ground biomass. Additionally, decreasing radial growth of *P. sylvestris* with increasing temperature (Martínez-Vilalta et al., 2008; Michelot et al., 2012) could also contribute to lower above-ground biomass in the greenhouse compared to the vegetation hall.

The seasonal drought treatments (spring and/or summer drought) in 2013 did not affect dimensions measured in 2015. Most likely, effects induced by the drought treatment in 2013 were diluted since potting seedlings into much larger pots at the beginning of March 2014 caused an increase in overall mean above-ground dry weight by a factor of almost 6 (27.0 g in 2014 experiment, 156.4 g in 2015 experiment).

Nevertheless, the reduction of above-ground biomass might be an adaptation linked to drought tolerance, either across provenances or caused by acclimation (temperature, water availability).The reduction of above-ground biomass is linked to a decrease of foliage (Xiao and Ceulemans, 2004; Jagodziński and Kałucka, 2008) which is reducing evaporative water loss (DeLucia et al., 2000). Future work should also consider molecular and physiological acclimation pathways which might be associated with provenance.

### Mortality Hazard Rates

Meteorological conditions during the 2015 mortality experiment were more severe than in the 2014 experiment, thus considerable first die-backs were observed after ∼60 and ∼40 days of completely withholding water, respectively.

It is difficult to disentangle acclimation effects (drought treatment 2014 and growing pines under different temperature regimes) as well as provenance effects on mortality hazard rates from pure dimension effects (above-ground biomass and height) because both acclimation treatments and provenance influenced above-ground dimensions (**Table 2**, **Supplementary Tables S1** and **S2**). We performed all survival modeling twice, with absolute dimensions (not reported in the paper) and with dimensions centered on respective means of acclimation treatments and provenances. In the latter case dimension effects were merely effects on the deviations from any provenance effect, and thus independent thereof. Since both attempts led to similar results, we conclude that (1) besides provenance and acclimation sheer above-ground dry weight (2014 experiment) and weight and height (2015 experiment) variations within provenances and treatments influenced mortality hazard (as presented in the Section “Result”) and that (2) the provenance induced dimension differences were not the only provenance-treatment effects, as even by accounting for absolute differences in dimensions, there was still an additional provenance/acclimation effect, likely linked to physiological, wood anatomical differences, and/or adjustments.

#### Provenance Effects on Mortality Hazard Rates

Drought mortality was fundamentally different across provenances in both experiments, thus fully supporting our first hypothesis. This finding is novel since Cregg and Zhang (2001) could not find any variation in mortality rates of *P. sylvestris* seedlings from several Eastern European and Central Asian origins.

Opposite to our expectations, we could not identify any clear continuous relationship between provenance survival and the climate at their origin, such as mean annual temperature and mean annual precipitation sum (**Table 1**, data of correlation analysis not shown). However, the three provenances withstanding drought mortality the most (Emilia Romagna, Prealpes du Sud, Garmen) originate from locations with a pronounced precipitation minimum in summer, whereas the three provenances which were at the highest risk of drought mortality (Alpenkiefer, Hauptsmoorwald, Wallis) experience relatively high precipitation at their origin throughout the year (**Supplementary Figure S1**). *F. sylvatica* provenances originating from locations with summer drought had lower mortality rates under experimental induced extreme drought than provenances from wetter locations (Thiel et al., 2014). In contrast to their study, all the Scots pine provenances used in our study are from its south-western distribution range, which might blur an obvious drought survival–climate relationship. Mortality rates in *P. ponderosa* could not been related to climatic variables either (Cregg, 1994). Rather to show a distinct pattern between mortality and climate, Ponderosa pine seems to have an optimum root–shoot ratio balancing water accessibility and photosynthetic capacity.

#### Trade-Off between Growth and Drought Survival

The hazard of mortality significantly varied with above-ground dimensions, a fact that was not explicitly mentioned in our hypotheses but was subsumed in the variable provenance. The mortality hazard increased with above ground dimensions suggesting a trade-off between growth and drought survival. In dry years, Alía et al. (2001) observed a lower branch die-off on slow growing provenances than on faster growing ones. On a global scale, larger trees have higher mortality rates than smaller ones (Bennett et al., 2015). Since taller trees face greater challenges to lift water along height against gravity and conduit resistance, they are at higher risk of hydraulic dysfunction than smaller trees (McDowell and Allen, 2015). In neotropical savannas, larger trees lose 50% of hydraulic conductivity at higher values of water potential than smaller tress (Zhang et al., 2009). Taller trees counteract this hydraulic limitation by reducing stomatal conductance and leaf specific hydraulic conductance (Ryan et al., 2006) and since needle area is positively correlated with tree height and above-ground biomass (Xiao and Ceulemans, 2004; Jagodziński and Kałucka, 2008), this increases the hazard for hydraulic failure of larger trees (McDowell and Allen, 2015). Additionally, individuals with a larger transpiring needle area might exploit water resources faster than smaller individuals and thus die earlier under severe drought conditions.

#### Acclimation Reduces the Hazard of Drought Induced Mortality

Although we did not measure acclimation directly we suppose that exposure time to the treatments was sufficient since thermal acclimation can occur within hours to few days (Mäkelä et al., 2004; Lee et al., 2005). In addition, we could observe a change of above-ground dimensions caused by temperature and drought acclimation treatments in our study.

The drought acclimation treatment in 2014 significantly decreased mortality hazard rates as suggested in our second hypothesis. However, acclimation effects of 2013, two years prior to the mortality treatment, could not be identified any more, most likely due to the re-potting, significantly increasing seedlings’ size. It might be worth to redo such a multiple acclimation mortality experiment without this confounding effect. Our finding of an acclimation effect in drought-induced mortality is in accordance to drought preconditioned Ponderosa pine seedlings which survived 2 weeks longer during drought than untreated seedlings and might be related to reduced gas exchange caused by stomatal control of water loss (Cregg, 1994).

It is known from literature that drought reduces needle area, decreases leaf/sapwood area ratio, and/or leads to smaller conduit sizes in Scots pine (Sterck et al., 2008; Martínez-Vilalta et al., 2009). Adjustments like these, might have favored higher survival rates under severe drought conditions in our study since they support avoidance of critical water potentials: Smaller leaf area results in lower transpiration rates reducing soil water exploitation (Verbeeck et al., 2007), decrease of leaf/sapwood area ratio results in total leaf area to be supported by a relatively larger area of water conducting tracheids (Mencuccini and Grace, 1995), and smaller conduit sizes increase resistance to cavitation (Sterck et al., 2012). Lower above-ground biomass and height of drought acclimated individuals in our study might also be linked to lower total needle area and thus preserves water transport and soil water resources by decreasing the risk of hydraulic failure and lower transpiration rates respectively. A drought induced decrease in leaf/sapwood area might be reflected by lower above-ground biomass in our study possible driven by lower needle biomass resulting in smaller total needle area.

The provenance-specific temperature effect on the hazard of mortality might be driven by changes in dimensions since variations in above-ground biomass follow the same pattern as hazard of mortality between provenances and buildings (data not shown) when only three provenances were considered in the mortality experiment 2015. As discussed above, elevated temperatures decrease height and diameter growth in Scots pine (Martínez-Vilalta et al., 2008; Reich and Oleksyn, 2008; Michelot et al., 2012). Additionally, high temperatures increase VPD, which was equally observed in our study. An increase of VPD alters biomass allocation reducing relative investment in leaf biomass (DeLucia et al., 2000), which in turn might decrease the hazard of mortality by a reduction of the transpiring surface.

However, it is also reported in the literature that exposure of seedlings to high temperatures can increase thermal tolerance caused by expression of heat shock proteins (Colombo and Timmer, 1992). Unfortunately little is known about their “life-time” and thus it is unclear whether pines grown in the greenhouse and the vegetation hall from 2012 to 2014 still expressed different levels of heat shock proteins after growing exclusively in the greenhouse from December 11, 2014 till the start of the mortality experiment on May 22, 2015. The fact that in our study provenance effects were only apparent in the high temperature regime might suggest some association with heat shock proteins, however, this needs to be thoroughly tested in a follow-up study.

## Conclusion

Above-ground dimensions were the main determinants of seedling mortality in Scots pine. Conversely, the pattern of provenances’ specific hazards for drought mortality did not follow the pattern of above-ground biomass when 12 provenances were studied. Thus, also adaptations to local climate and genetic specific control of water relations (e.g., transpiration and stomatal conductance), which was observed in *P. ponderosa* and *Pinus taeda* (Seiler and Johnson, 1988; Monson and Grant, 1989), might play a dominating role in resistance to drought mortality. Our study revealed a clear acclimation potential of Scots Pine seedlings, since drought episodes and warmer temperatures increased their survival time under repeated stresses. This feature might facilitate Scots pine forest persistence under future climate change in the long run.

## Author Contributions

HS collected data, contributed to the experimental design, analyzed and interpreted the data, and wrote the paper. AM contributed to the conception of the work and experimental design, interpreted the data, and wrote the paper.

## Conflict of Interest Statement

The authors declare that the research was conducted in the absence of any commercial or financial relationships that could be construed as a potential conflict of interest.
