# Supplementary material for: Above-Ground Dimensions and Acclimation Explain Variation in Drought Mortality of Scots Pine Seedlings from Various Provenances
Source: Front Plant Sci. 2016 Jul 7;7:1014. doi: 10.3389/fpls.2016.01014 (PMC4935725; doi:10.3389/fpls.2016.01014)
Supplement: Supplementary file 1 [file Data_Sheet_1.DOCX]

Supplementary Material

Size and acclimation modify drought mortality of Scots pine seedlings from various provenances

Hannes Seidel*, Annette Menzel

*** Correspondence:** Hannes Seidel: hseidel@wzw.tum.de

# Supplementary Data

# Supplementary Figures and Tables

## Supplementary Figures


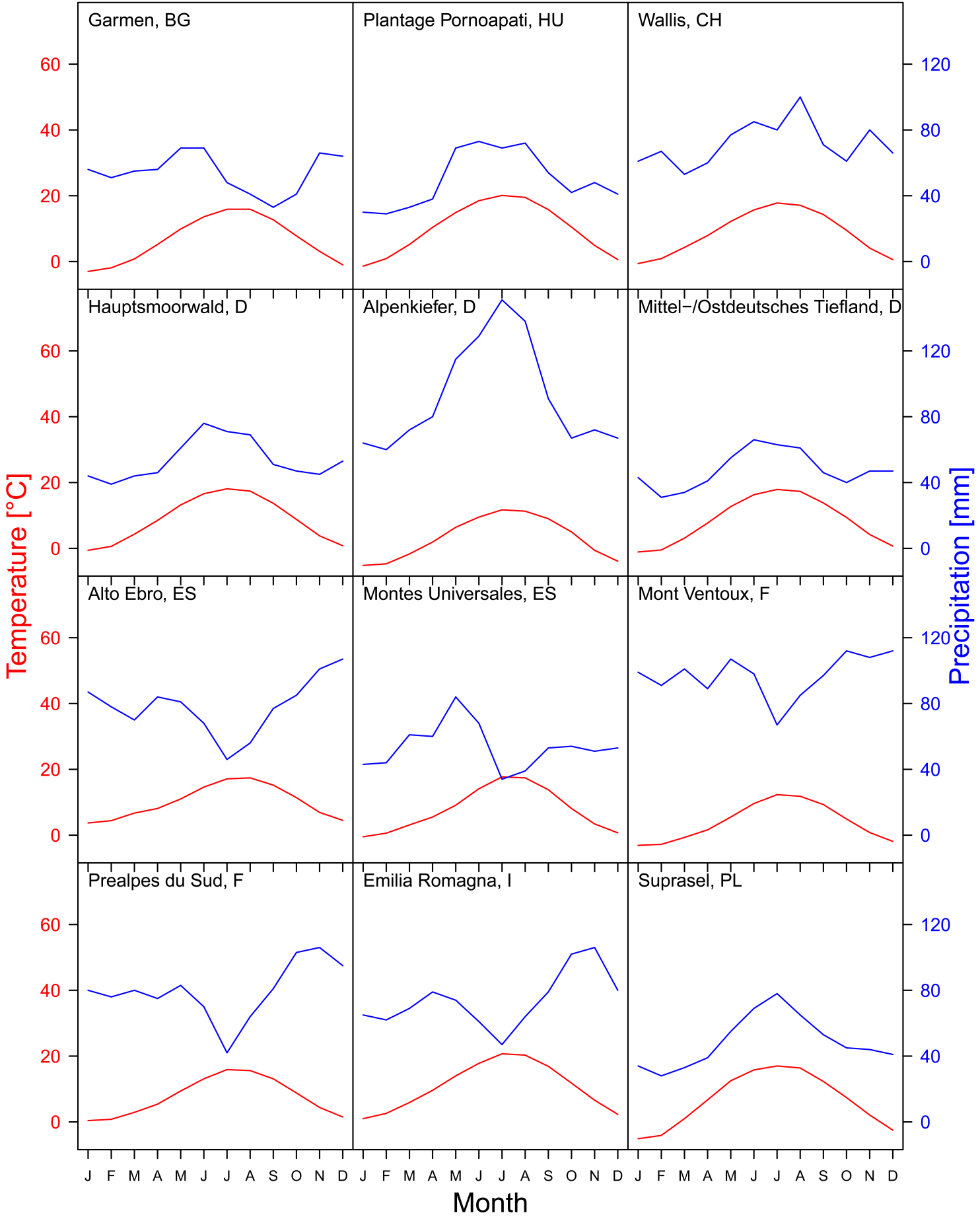


**Supplementary Figure 1. Climatograms at the origin of provenances.** Climate data obtained from the WorldClim data base (Hijmans et al., 2005) for the period 1950 – 2000.


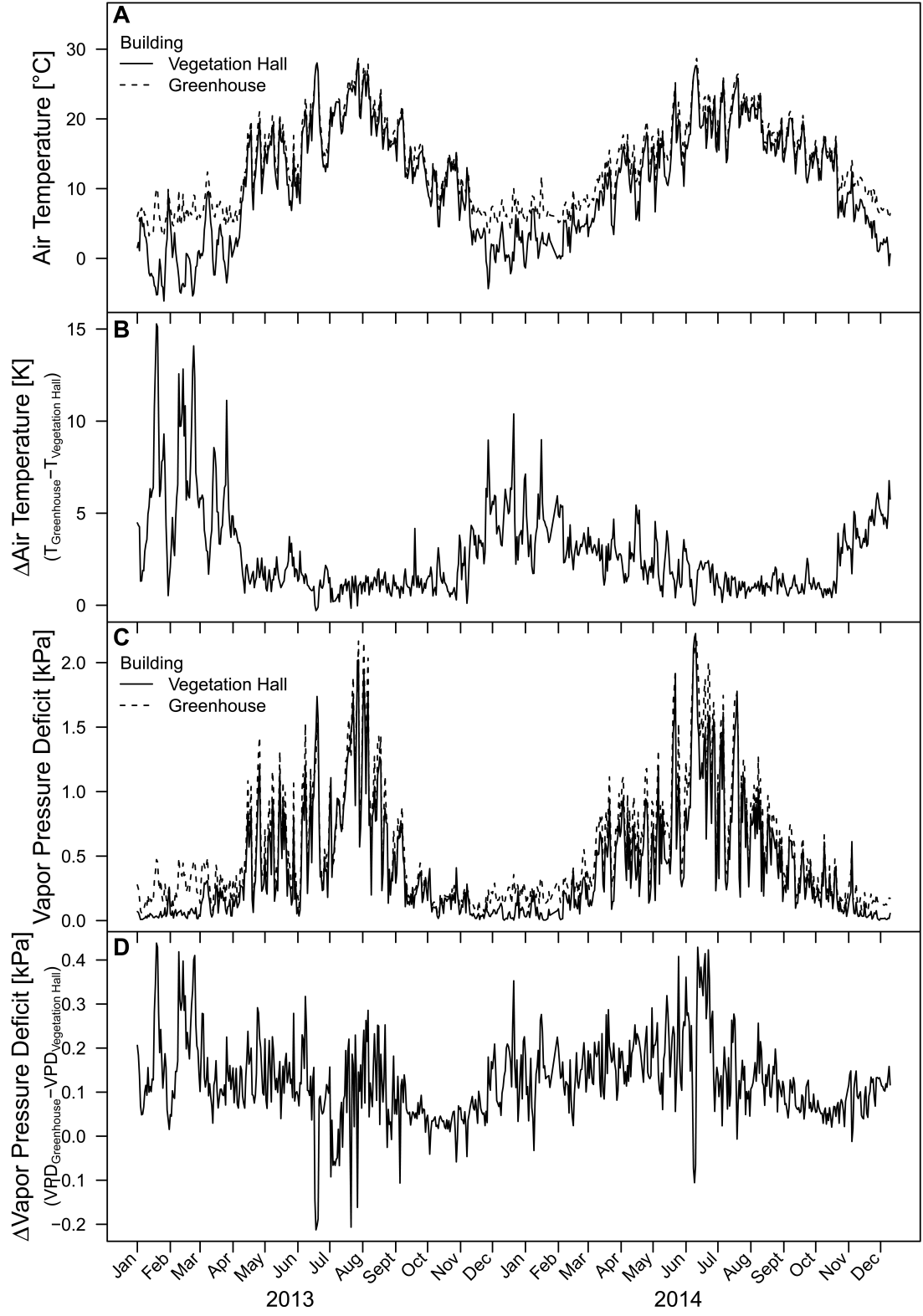


**Supplementary Figure 2. Temperature acclimation treatment and vapor pressure deficit experienced by the Scots pine seedlings used in the mortality experiment in 2015.** (A) Mean daily air temperature (T) in the vegetation hall and the greenhouse. (B) Differences of mean daily air temperatures (T) between the greenhouse and vegetation hall. (C) Mean daily vapor pressure deficit (VPD) in the vegetation hall and the greenhouse and (D) differences of mean daily vapor pressure deficit (VPD) between the greenhouse and vegetation hall.


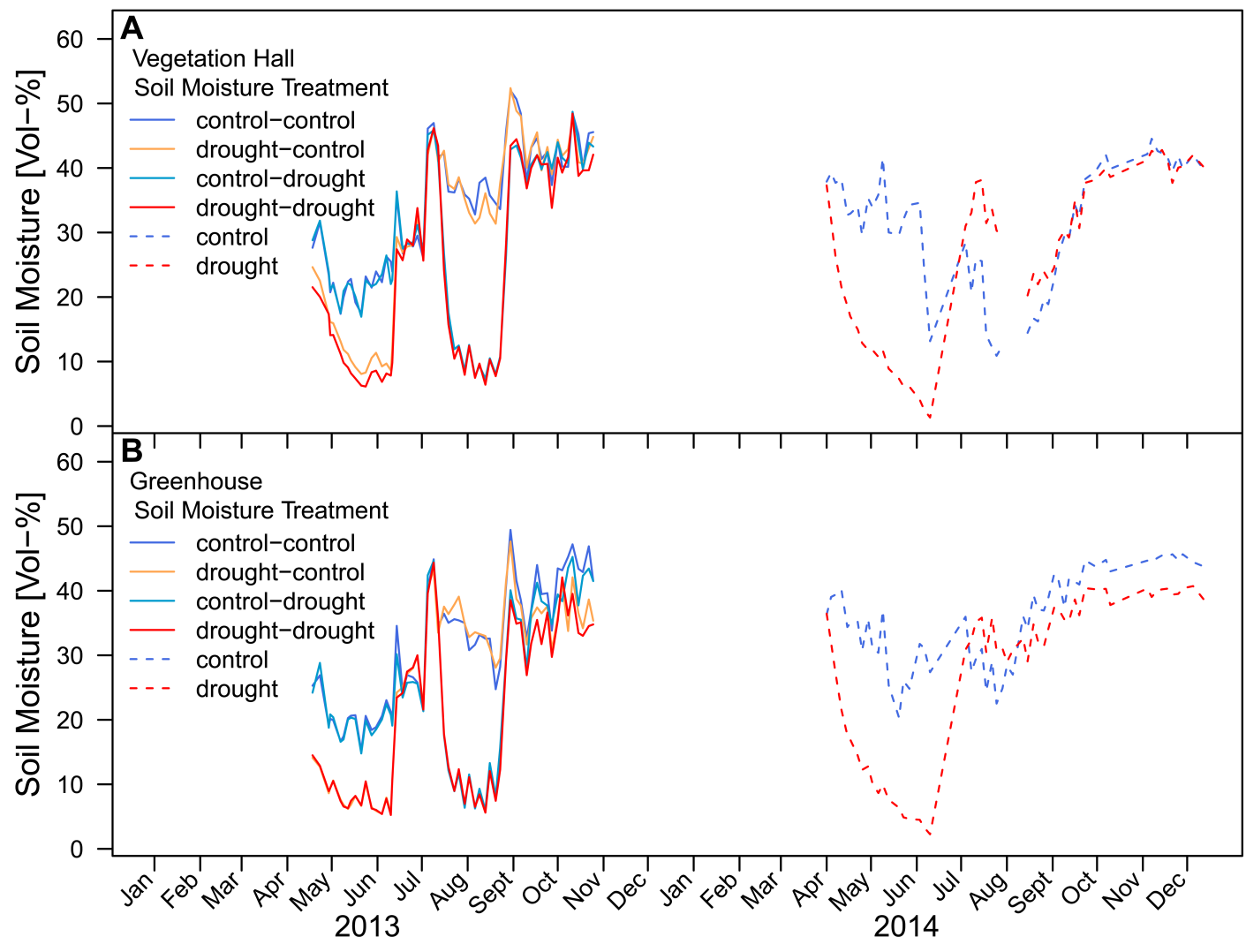


**Supplementary Figure 3. Drought acclimation treatments in 2013 and 2014 experienced by the Scots pine seedlings used in the mortality experiment in 2015.** (A) Soil moisture in the vegetation hall and (B) the greenhouse. Note that due to malfunction of the irrigation system in the vegetation hall in July and August 2014, soil moisture of the control treatment was partially lower than in the drought treatment.


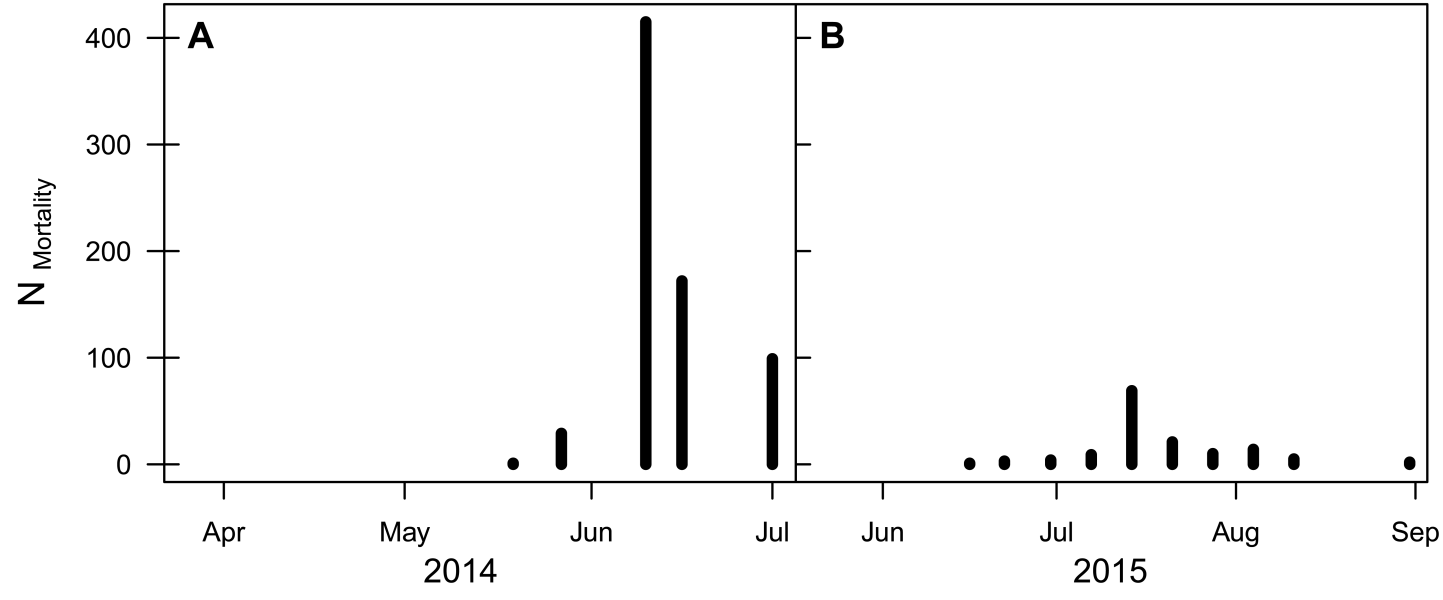


**Supplementary Figure 4. Observed mortality events during the experiments in (A) 2014 and (B) 2015.**

## Supplementary Tables

**Supplementary Table 1. Summary of the linear model evaluating the influence of provenance on seedling above-ground dry weight.** Estimates are calculated in reference to the intercept (in this case BG10). Provenance abbreviations are BG10 (Garmen, BG), HU14 (Plantage Pornoapati, HU), CH5 (Wallis, CH), D6 (Hauptsmoorwald, D), D7 (Alpenkiefer, D), D8 (Mittel-/Ostdt. Tiefland, D), ES1 (Alto Ebro, ES), ES2 (Montes Universales, ES), F12 (Mont Ventoux, F), F3 (Prealpes du Sud, F), I4 (Emilia Romagna, I) and PL9 (Suprasl, PL).

|  | Weight ± SD | t | p |
| --- | --- | --- | --- |
| (Intercept) | 30.712 ± 1.124 | 27.326 | **<0.001** |
| CH5 | -8.326 ± 1.389 | -5.995 | **<0.001** |
| D6 | -2.669 ± 1.806 | -1.478 | 0.140 |
| D7 | 4.105 ± 1.574 | 2.608 | **0.009** |
| D8 | -0.254 ± 1.677 | -0.151 | 0.880 |
| ES1 | -5.047 ± 1.539 | -3.279 | **0.001** |
| ES2 | -7.383 ± 1.265 | -5.838 | **<0.001** |
| F12 | -3.297 ± 1.533 | -2.151 | **0.032** |
| F3 | -4.144 ± 1.606 | -2.580 | **0.010** |
| HU14 | 1.072 ± 1.574 | 0.681 | 0.496 |
| I4 | -0.125 ± 1.654 | -0.075 | 0.940 |
| PL9 | -3.099 ± 1.590 | -1.950 | 0.052 |
| Adjusted R^2^ = 0.1763, p < 0.001 | | | |

**Supplementary Table 2. Summary of the single linear models evaluating the influence of provenance and acclimation treatments (drought treatment 2013, drought treatment 2014 and building) on seedling above-ground dry weight and height.** Estimates are calculated in reference to the intercepts, that are D7 (Provenance), drought-drought, meaning spring and summer drought (drought treatment 2013), drought (drought treatment 2014) and greenhouse (building). Provenance abbreviations are D7 (Alpenkiefer, D), F12 (Mont Ventoux, F), PL9 (Suprasl, PL). Group differences were calculated comparing contrasts with the Tukey´s range test

|  | Weight ± SD [g] | t | p |  | Height ± SD [mm] | t | p |
| --- | --- | --- | --- | --- | --- | --- | --- |
| Provenance |  |  |  |  |  |  |  |
| (Intercept) | 182.23 ± 7.33 | 24.85 | **<0.001** |  | 956.19 ± 30.36 | 31.48 | **<0.001** |
| F12 | -48.30 ± 10.31 | -4.69 | **<0.001** |  | -221.35 ± 42.69 | -5.19 | **<0.001** |
| PL9 | -28.36 ± 9.96 | -2.85 | **0.005** |  | 74.66 ± 41.03 | 1.82 | 0.071 |
| Adjusted R^2^ = 0.128, p = **<0.001** | | | |  | Adjusted R^2^ = 0.279, p = **<0.001** | | |
| Drought treatment 2013 | |  |  |  |  |  |  |
| (Intercept) | 145.11 ± 9.09 | 15.97 | **<0.001** |  | 865.44 ± 40.65 | 21.29 | **<0.001** |
| Drought-control | 11.53 ± 12.49 | 0.92 | 0.357 |  | -2.63 ± 55.51 | -0.05 | 0.962 |
| Control-drought | 8.79 ± 12.66 | 0.69 | 0.489 |  | 57.77 ± 56.64 | 1.02 | 0.310 |
| Control-control | 23.28 ± 12.49 | 1.86 | 0.065 |  | 135.84 ± 55.87 | 2.43 | **0.016** |
| Adjusted R^2^ = 0.004, p = 0.314 | | | |  | Adjusted R^2^ = 0.038, p = **0.041** | | |
| Drought treatment 2014 | |  |  |  |  |  |  |
| (Intercept) | 131.49 ± 4.75 | 27.66 | **<0.001** |  | 858.9 ± 25.11 | 34.2 | **<0.001** |
| Control | 59.15 ± 7.33 | 8.07 | **<0.001** |  | 132.17 ± 38.88 | 3.4 | **<0.001** |
| Adjusted R^2^ = 0.320, p = **<0.001** | | | |  | Adjusted R^2^ = 0.071, p = **<0.001** | | |
| Building |  |  |  |  |  |  |  |
| (Intercept) | 141.87 ± 5.73 | 24.77 | **<0.001** | | 826.05 ± 25.05 | 32.97 | **<0.001** |
| Vegetation hall | 31.23 ± 8.41 | 3.71 | **<0.001** | | 188.18 ± 36.64 | 5.14 | **<0.001** |
| Adjusted R^2^ = 0.085, p = **<0.001** | | | |  | Adjusted R^2^ = 0.155, p = **<0.001** | | |

**Supplementary Table 3. Summary of the Cox proportional hazards regression model evaluating mortality in the mortality experiment 2014 showing model coefficient and standard error (SE), hazard ratio (HR) and 95% confidence interval ([95% CI]), z-score and p-value.** Hazard ratios are calculated in reference to the base hazard of D7. Provenance abbreviations are BG10 (Garmen, BG), HU14 (Plantage Pornoapati, HU), CH5 (Wallis, CH), D6 (Hauptsmoorwald, D), D7 (Alpenkiefer, D), D8 (Mittel-/Ostdt. Tiefland, D), ES1 (Alto Ebro, ES), ES2 (Montes Universales, ES), F12 (Mont Ventoux, F), F3 (Prealpes du Sud, F), I4 (Emilia Romagna, I) and PL9 (Suprasl, PL).

|  | Coefficient ± SE | HR [95% CI] | | z | p |
| --- | --- | --- | --- | --- | --- |
| CH5 | -0.325 ± 0.177 | 0.722 [0.511 - 1.021] | | -1.841 | 0.066 |
| F3 | -1.002 ± 0.214 | 0.367 [0.241 - 0.559] | | -4.680 | **<0.001** |
| F12 | -0.489 ± 0.195 | 0.613 [0.418 - 0.899] | | -2.504 | **0.012** |
| ES1 | -0.489 ± 0.197 | 0.613 [0.417 - 0.902] | | -2.481 | **0.013** |
| I4 | -1.409 ± 0.226 | 0.244 [0.157 - 0.380] | | -6.246 | **<0.001** |
| BG10 | -0.940 ± 0.208 | 0.391 [0.260 - 0.587] | | -4.515 | **<0.001** |
| D6 | -0.219 ± 0.231 | 0.803 [0.510 - 1.264] | | -0.948 | 0.343 |
| ES2 | -0.385 ± 0.161 | 0.681 [0.496 - 0.933] | | -2.388 | **0.017** |
| HU14 | -0.441 ± 0.201 | 0.643 [0.434 - 0.953] | | -2.200 | **0.028** |
| PL9 | -0.402 ± 0.204 | 0.669 [0.449 - 0.997] | | -1.975 | **0.048** |
| D8 | -0.743 ± 0.219 | 0.476 [0.310 - 0.730] | | -3.396 | **0.001** |
| weight | 0.017 ± 0.004 | 1.017 [1.008 - 1.026] | | 3.857 | **<0.001** |
|  |  |  |  |  |  |
| Concordance = 0.659 ± 0.026 | | | | | |
| R^2^ = 0.109 | | | | | |
| Likelihood ratio test = 86.18 on 12 df, p = 2.7e^-13^ | | | | | |
| Wald test = 78.53 on 12 df, p = 7.878e^-12^ | | | | | |
| Score (logrank) test = 81.94 on 12 df, p = 1.755e^-12^ | | | | | |

**Supplementary Table 4. Estimated effects of the survival model on the hazard for mortality of the experiment in 2014.** The hazard ratio (HR) shows the change of hazard in relation to the change in above-ground dry weight per gram (weight (ΔHR/g)) and the hazard between pairs of provenances. Provenance abbreviations are BG10 (Garmen, BG), HU14 (Plantage Pornoapati, HU), CH5 (Wallis, CH), D6 (Hauptsmoorwald, D), D7 (Alpenkiefer, D), D8 (Mittel-/Ostdt. Tiefland, D), ES1 (Alto Ebro, ES), ES2 (Montes Universales, ES), F12 (Mont Ventoux, F), F3 (Prealpes du Sud, F), I4 (Emilia Romagna, I) and PL9 (Suprasl, PL). Group differences were calculated comparing contrasts with the Tukey´s range test.

| Rate of change in hazard | |  |  |  |  |  |
| --- | --- | --- | --- | --- | --- | --- |
|  | HR | p |  |  |  |  |
| Weight (ΔHR/g) | 1.017 | <0.001 |  |  |  |  |
| Pairwise comparisons of provenances | | |  |  |  |  |
|  | HR | p |  |  | HR | p |
| D7 / CH5 | 1 /0.723 | 0.120 |  | F12 / D6 | 1 /1.31 | 0.363 |
| D7 / F3 | 1 /0.367 | **<0.001** |  | F12 / ES2 | 1 /1.111 | 0.626 |
| D7 / F12 | 1 /0.613 | **0.037** |  | F12 / HU14 | 1 /1.049 | 0.847 |
| D7 / ES1 | 1 /0.613 | **0.037** |  | F12 / PL9 | 1 /1.091 | 0.747 |
| D7 / I4 | 1 /0.244 | **<0.001** |  | F12 / D8 | 1 /0.776 | 0.363 |
| D7 / BG10 | 1 /0.391 | **<0.001** |  | ES1 / I4 | 1 /0.399 | **<0.001** |
| D7 / D6 | 1 /0.803 | 0.472 |  | ES1 / BG10 | 1 /0.637 | 0.061 |
| D7 / ES2 | 1 /0.68 | **0.041** |  | ES1 / D6 | 1 /1.31 | 0.363 |
| D7 / HU14 | 1 /0.643 | 0.061 |  | ES1 / ES2 | 1 /1.11 | 0.626 |
| D7 / PL9 | 1 /0.668 | 0.093 |  | ES1 / HU14 | 1 /1.049 | 0.847 |
| D7 / D8 | 1 /0.476 | **0.003** |  | ES1 / PL9 | 1 /1.091 | 0.747 |
| CH5 / F3 | 1 /0.508 | **0.002** |  | ES1 / D8 | 1 /0.776 | 0.363 |
| CH5 / F12 | 1 /0.849 | 0.472 |  | I4 / BG10 | 1 /1.598 | 0.081 |
| CH5 / ES1 | 1 /0.849 | 0.472 |  | I4 / D6 | 1 /3.284 | **<0.001** |
| CH5 / I4 | 1 /0.339 | **<0.001** |  | I4 / ES2 | 1 /2.784 | **<0.001** |
| CH5 / BG10 | 1 /0.541 | **0.004** |  | I4 / HU14 | 1 /2.633 | **<0.001** |
| CH5 / D6 | 1 /1.112 | 0.738 |  | I4 / PL9 | 1 /2.735 | **<0.001** |
| CH5 / ES2 | 1 /0.943 | 0.747 |  | I4 / D8 | 1 /1.946 | **0.019** |
| CH5 / HU14 | 1 /0.89 | 0.626 |  | BG10 / D6 | 1 /2.056 | **0.009** |
| CH5 / PL9 | 1 /0.926 | 0.747 |  | BG10 / ES2 | 1 /1.742 | **0.005** |
| CH5 / D8 | 1 /0.658 | 0.069 |  | BG10 / HU14 | 1 /1.647 | **0.041** |
| F3 / F12 | 1 /1.67 | **0.037** |  | BG10 / PL9 | 1 /1.713 | **0.033** |
| F3 / ES1 | 1 /1.67 | **0.038** |  | BG10 / D8 | 1 /1.218 | 0.508 |
| F3 / I4 | 1 /0.666 | 0.145 |  | D6 / ES2 | 1 /0.848 | 0.532 |
| F3 / BG10 | 1 /1.064 | 0.841 |  | D6 / HU14 | 1 /0.801 | 0.472 |
| F3 / D6 | 1 /2.188 | **0.005** |  | D6 / PL9 | 1 /0.833 | 0.561 |
| F3 / ES2 | 1 /1.853 | **0.003** |  | D6 / D8 | 1 /0.592 | 0.069 |
| F3 / HU14 | 1 /1.752 | **0.028** |  | ES2 / HU14 | 1 /0.945 | 0.799 |
| F3 / PL9 | 1 /1.822 | **0.019** |  | ES2 / PL9 | 1 /0.982 | 0.929 |
| F3 / D8 | 1 /1.296 | 0.385 |  | ES2 / D8 | 1 /0.699 | 0.093 |
| F12 / ES1 | 1 /1 | 1 |  | HU14 / PL9 | 1 /1.04 | 0.875 |
| F12 / I4 | 1 /0.399 | **<0.001** |  | HU14 / D8 | 1 /0.739 | 0.281 |
| F12 / BG10 | 1 /0.637 | 0.060 |  | PL9 / D8 | 1 /0.711 | 0.213 |

**Supplementary Table 5. Summary of the Cox proportional hazards regression model evaluating mortality in the mortality experiment 2015 for the factorial model showing model coefficient and standard error (SE), hazard ratio (HR) and 95% confidence interval ([95% CI]), z-score and p-value.** Hazard ratios are calculated in reference to the base hazard, in this case in regard to D7 in the control treatment in 2014 and grown in the vegetation hall till December 2014. Provenance abbreviations are D7 (Alpenkiefer, D), PL9 (Surprasl, PL) and F12 (Mont Ventoux, F).

|  | Coefficient ± SE | HR [95% CI] | | z | p |
| --- | --- | --- | --- | --- | --- |
| Drought treatment 2014 dry | -0.854 ± 0.194 | 0.426 [0.291 - 0.623] | | -4.399 | **<0.001** |
| Provenance F12 | 0.081 ± 0.328 | 1.085 [0.570 - 2.064] | | 0.248 | 0.804 |
| Provenance PL9 | 0.301 ± 0.298 | 1.351 [0.754 - 2.422] | | 1.011 | 0.312 |
| Building greenhouse | -0.035 ± 0.312 | 0.965 [0.524 - 1.778] | | -0.114 | 0.909 |
| Provenance F12 :  building greenhouse | -1.023 ± 0.452 | 0.360 [0.148 - 0.872] | | -2.264 | **0.024** |
| Provenance PL9 :  building greenhouse | -0.985 ± 0.427 | 0.373 [0.162 - 0.862] | | -2.309 | **0.021** |
|  |  |  |  |  |  |
| Concordance = 0.738 ± 0.05 | | | | | |
| R^2^ = 0.271 | | | | | |
| Likelihood ratio test = 43.92 on 6 df, p = 7.668e^-08^ | | | | | |
| Wald test = 43.08 on 6 df, p = 1.126e^-07^ | | | | | |
| Score (logrank) test = 45.38 on 6 df, p = 3.937e^-08^ | | | | | |

**Supplementary Table 6. Summary of the Cox proportional hazards regression model evaluating mortality in the mortality experiment 2015 for the continuous model showing model coefficient and standard error (SE), hazard ratio (HR) and 95% confidence interval ([95% CI]), z-score and p-value.** Hazard ratios are calculated in reference to the base hazard, in this case in regard to D7 in the control treatment in 2014 and grown in the vegetation hall. Provenance abbreviations are D7 (Alpenkiefer, D), PL9 (Surprasl, PL) and F12 (Mont Ventoux, F).

|  | Coefficient ± SE | HR [95% CI] | z | p |
| --- | --- | --- | --- | --- |
| Height | 0.002 ± 0.001 | 1.002 [1.001 - 1.003] | 2.811 | **0.005** |
| Weight | 0.008 ± 0.003 | 1.008 [1.002 - 1.014] | 2.759 | **0.006** |
| Drought treatment 2014 dry | -0.907 ± 0.200 | 0.404 [0.273 - 0.597] | -4.541 | **<0.001** |
| Provenance F12 | 0.370 ± 0.337 | 1.447 [0.747 - 2.802] | 1.096 | 0.273 |
| Provenance PL9 | 0.410 ± 0.305 | 1.506 [0.828 - 2.739] | 1.342 | 0.180 |
| Building greenhouse | 0.166 ± 0.322 | 1.181 [0.628 - 2.219] | 0.515 | 0.606 |
| Provenance F12 :  building greenhouse | -1.495 ± 0.47 | 0.224 [0.089 - 0.563] | -3.182 | **0.001** |
| Provenance PL9 :  building greenhouse | -1.037 ± 0.437 | 0.355 [0.151 - 0.835] | -2.374 | **0.018** |
|  |  |  |  |  |
| Concordance = 0.806 ± 0.05 | | | | |
| R^2^ = 0.397 | | | | |
| Likelihood ratio test = 69.84 on 8 df, p=5.281e^-12^ | | | | |
| Wald test = 67.05 on 8 df, p=1.899e^-11^ | | | | |
| Score (logrank) test = 71.2 on 8 df, p=2.834e^-12^ | | | | |

## Supplementary References

Hijmans, R. J., Cameron, S. E., Parra, J. L., Jones, P. G., and Jarvis, A. (2005). Very high resolution interpolated climate surfaces for global land areas. *Int. J. Climatol.* 25, 1965–1978. doi:10.1002/joc.1276.
